# Supplementary material for: Occurrence of Aflatoxins in Edible Vegetable Seeds and Oil Samples Available in Pakistani Retail Markets and Estimation of Dietary Intake in Consumers
Source: Int J Environ Res Public Health. 2021 Jul 29;18(15):8015. doi: 10.3390/ijerph18158015 (PMC8345775; doi:10.3390/ijerph18158015)
Supplement: Supplementary file 1 [file ijerph-18-08015-s001.zip › ijerph-1133564-supplementary.pdf]

Sunflower oil and seeds

Branded oil			Unbranded oil			Branded seeds			Unbranded seeds		
**SR. #**	**AFB1**	**TAFs**	**SR.#**	**AFB1**	**TAFs**	**SR. #**	**AFB1**	**TAFs**	**SR.#**	**AFB1**	**TAFs**
1	4.50	9.10	1	10.5	13.5	1	4.3	11.3	1	6.7	18.5
2	7.80	13.5	2	9.6	14.5	2	6.5	18.9	2	6.8	21.5
3	3.50	11.70	3	2.5	13.5	3	5.7	20.6	3	8.5	22.4
4	6.78	12.50	4	7.7	14.5	4	6.5	13.6	4	8.5	15.5
5	15.80	25.70	5	22.8	28.9	5	123.5	140.7	5	125.5	148.6
6	37.70	45.6	6	41.6	49.6	6	36.5	38.9	6	38.5	42.5
7	14.6	25.7	7	19.6	28.7	7	21.4	21.9	7	25.4	27.5
8	8.9	35.8	8	9.10	38.9	8	5.6	14.6	8	6.5	15.5
9	6.5	11.5	9	8.9	12.5	9	7.6	19.6	9	7.5	21.5
10	2.4	9.8	10	3.6	14.5	10	7.3	13.5	10	8.5	14.5
11	3.5	15.6	11	6.7	22.6	11	5.7	11.6	11	6.5	14.5
12	6.7	15.7	12	2.4	11.5	12	10.6	21.6	12	12.5	22.5
			13	3.6	18.9	13	22.6	29.7	13	25.5	32.5
			14	2.5	11.5	14	16.7	26.9	14	18.5	29.5
			15	2.4	21.6	15	22.5	33.5	15	25.4	36.5
						16	15.6	34.6	16	18.5	39.5
						17	9.23	24.6	17	10.5	26.5
						18	4.5	14.6	18	5.5	15.5
									19	8.5	11.4
									20	9.4	15.5

Soyabean oil and seeds

Branded oil			Unbranded oil			Branded seeds			Unbranded seeds		
**SR. #**	**AFB1**	**TAFs**	**SR.#**	**AFB1**	**TAFs**	**SR. #**	**AFB1**	**TAFs**	**SR.#**	**AFB1**	**TAFs**
1	4.5	6.5	1	5.6	7.8	1	6.8	11.5	1	7.8	12.5
2	12.5	20.5	2	13.5	25.6	2	47.8	67.9	2	50.4	69.8
3	3.4	10.5	3	4.5	12.4	3	5.6	15.6	3	5.4	11.3
4	2.5	14.6	4	3.5	16.5	4	4.6	19.5	4	5.7	20.5
5	34.6	94.5	5	44.5	95.6	5	48.6	93.5	5	54.6	98.5
6	45.6	54.6	6	34.5	44.5	6	36.5	57.8	6	34.6	58.7
7	2.5	6.7	7	4.5	9.6	7	5.6	10.5	7	6.5	14.5
8	8.9	9.5	8	11.5	23.5				8	7.6	23.5
9	10.5	11.5	9	6.7	21.4				9	9.6	21.5
			10	5.6	12.3						

Canola oil and seeds

Branded oil			Unbranded oil			Branded seeds			Unbranded seeds		
**SR. #**	**AFB1**	**TF**	**SR.#**	**AFB1**	**TF**	**SR. #**	**AFB1**	**TF**	**SR.#**	**AFB1**	**TF**
1	3.4	4.5	1	4.6	9.8	1	6.5	11.3	1	7.65	15.5
2	6.7	7.5	2	9.8	16.7	2	10.5	19.6	2	15.5	20.5
3	4.5	11.4	3	5.6	17.5	3	5.7	20.5	3	6.5	22/4
4	45.6	86.7	4	51.4	91.5	4	61.5	110.5	4	65.5	112.5
5	2.5	10.5	5	3.5	13.6	5	4.5	15.4	5	5.5	18.5
6	3.6	4.5	6	4.5	8.9	6	3.5	10.5	6	4.5	12.5
7	8.9	11.5	7	9.5	15.6	7	10.5	15.7	7	13.5	18.6
8	12.6	34.6	8	13.5	42.6	8	15.6	48.9	8	16.5	54.3
9	2.4	4.6	9	3.5	7.8	9	4,5	9.5	9	5.4	10.5
10	67.8	110.8	10	94.5	112.6	10	96.5	115.6	10	98.5	118.5
11	12.5	15.7	11	15.7	22.7	11	16.8	25.6	11	19.5	25.6
12	4.5	7.8	12	5.6	9.8	12	6.5	10.5	12	7.5	12.5
13	8.5	15.6	13	9.5	22.5	13	10.5	25.6	13	12.5	28.5
14	5.6	10.4	14	6.5	13.5	14	7.5	15.5	14	8.5	16.5
15	3.4	6.7	15	3.5	15.6				15	4.5	15.5
16	4.5	13.5	16	4.6	11.4				16	6.7	11.5
17	5.6	15.6	17	5.3	16.5				17	6.8	14.5
18	2.3	16.7	18	3.5	16.5				18	6.9	13.5
			19	5.6	11.4						
			20	2.4	8.5						
			21	3.5	6.5						
			22	4.5	8.5						

Olive oil and seeds

Branded oil			Unbranded oil			Branded seeds			Unbranded seeds		
**SR. #**	**AFB1**	**TAFs**	**SR.#**	**AFB1**	**TAFs**	**SR. #**	**AFB1**	**TF**	**SR.#**	**AFB1**	**TF**
1	2.4	5.6	1	3.5	6.7	1	3.4	6.7	1	6.7	11.5
2	3.5	9.8	2	4.5	11.5	2	4.5	10.5	2	6.9	15.7
3	2.4	11.8	3	3.4	13.5	3	4.6	7.6	3	9.6	15.7
4	34.5	58.9	4	39.8	65.6	4	45.7	81.9	4	55.7	86.7
5	37.8	44.5	5	42.5	58.7	5	56.8	76.5	5	58.9	78.9
6	2.4	7.6	6	3.4	9.8	6	3.4	9.5	6	4.5	6.7
7	2.5	4.5	7	3.5	6.5	7	3.5	6.5	7	7.8	13.5
8	2.3	6.7	8	2.6	7.8	8	4.5	9.5	8	5.6	14.5
			9			9			9	6.7	12.4
						10			10	2.4	4.5
						11					
						12					
						13					
						14					
						15					
						16					

Corn oil and seeds

Branded oil			Unbranded oil			Branded seeds			Unbranded seeds		
**SR. #**	**AFB1**	**TF**	**SR.#**	**AFB1**	**TF**	**SR. #**	**AFB1**	**TF**	**SR.#**	**AFB1**	**TF**
1	6.5	10.5	1	7.5	13.5	1	8.5	15.6	1	9.6	16.5
2	4.5	7.8	2	5.6	9.8	2	7.8	10.7	2	6.8	11.6
3	6.7	9.8	3	8.9	11.5	3	9.6	20.5	3	10.5	22.5
4	4.5	6.8	4	9.7	15.4	4	11.4	20.6	4	12.5	24.5

5	5.6	6.5	5	6.7	9.8	5	6.8	11.5	5	7.5	13.5
6	6.8	7.8	6	7.1	7.9	6	9.6	11.5	6	10.5	13.5
7	4.5	6.5	7	5.4	7.6	7	6.8	15.6	7	7.5	16.7
8	3.5	9.8	8	5.6	8.7	8	6.7	18.9	8	7.6	20.5
9	21.5	32.5	9	29.6	44.5	9	36.5	46.5	9	40.5	48.5
10	45.6	81.5	10	48.9	84.6	10	57.6	89.7	10	58.8	95.7
			11	22.4	29.7	11	26.7	36.5	11	28.6	47.8
			12	7.8	9.5	12	9.5	11.5	12	10.5	15.6
			13	2.5	11.5	13	3.5	6.7	13	5.6	7.8
			14	4.5	9.8	14	5.6	7.11	14	8.9	11.5
			15	34.6	45.6	15	49.7	65.6	15	57.8	87.5
			16	8.9	11.3	16	37.8	83.6	16	39.5	87.5
			17	2.4	4.5	17	2.5	7.8	17	4.5	9.7
			18	34.6	39.7	18	56.7	87.6	18	59.8	95.6
			19	3.5	6.7	19	2.5	7.8	21	3.5	9.7
			20	2.5	4.5	20	3.5	7.8	22	4.5	9.8
						21	7.8	9.4	23	9.5	12.4
						22	2.5	6.5	24	3.5	9.7
						23	4.5	6.7	25	5.6	7.8
						24	2.4	5.6	26	4.5	9.5
						25	9.8	11.4	27	10.5	15.5
									28	4.5	9.6
									29	3.5	10.5
									30	2.5	15.5

Mustard oil and seeds

**Branded oil**			**Unbranded oil**			**Branded seeds**			**Unbranded seeds**		
**SR. #**	**AFB1**	**TAFs**	**SR.#**	**AFB1**	**TAFs**	**SR. #**	**AFB1**	**TAFs**	**SR.#**	**AFB1**	**TAFs**
1	2.34	4.78	1	4.5	9.7	1	4.6	6.7	1	5.4	7.8
2	5.41	9.70	2	6.8	11.2	2	6.5	10.6	2	7.5	11.3
3	2.31	11.4	3	4.5	13.6	3	3.5	11.9	3	4.3	10.6
4	18.9	29.8	4	21.5	34.6	4	19.3	30.5	4	20.4	32.5
5	8.70	19.7	5	9.8	23.5	5	9.5	20.6	5	8.5	22.5
6	9.12	22.5	6	10.5	24.6	6	10.5	23.5	6	11.5	24.5
7	22.6	32.7	7	24.7	36.7	7	24.5	33.6	7	25.5	35.4
8	12.5	19.7	8	14.5	39.8	8	14.5	21.6	8	15.4	22.5
9	10.7	45.7	9	16.7	55.7	9	11.6	46.7	9	12.5	47.5
10	15.7	26.8	10	17.5	29.8	10	16.6	28.9	10	18.6	30.5
11	12.6	34.6	11	14.6	36.6	11	13.5	36.7	11	14.3	38.5
12	10.6	19.8	12	12.5	22.5	12	12.5	21.5	12	13.5	22.5
13	12.7	23.6	13	14.6	25.7	13	13.5	24.5	13	15.5	25.6
14	10.8	35.7	14	12.6	38.7	14	11.5	36.8	14	12.5	38.9
15	9.8	11.6	15	11.4	13.6	15	11.4	13.4	15	13.5	15.5
16	12.5	18.9	16	13.6	21.5	16	13.5	20.5	16	14.5	22.5
17	9.6	14.6	17	10.7	16.4	17	10.5	15.6	17	11.5	16.5
18	2.4	8.9	18	3.5	10.6	18	3.5	10.5	18	4.5	12.5
19	1.6	3.5	19	2.5	5.6	19	2.5	15.6	19	3.5	11.5
20	3.5	8.9	20	5.4	9.19	20	4.5	10.8	20	5.4	12.5
21	4.9	10.7	21	6.5	11.6				21	6.5	10.8
			22	7.9	16.8				22	7.8	34.6
			23	10.5	14.5				23	19.5	21.5
			24	11.4	18.9						
			25	12.5	16.5						
			26	10.5	14.5						
			27	9.5	10.6						
			28	4.5	8.6						

Food Frequency Questionnaire

Full Name	Age (y)	Sex
Weight (kg)	City	District
CNIN	Height (m)	Covid 19= Positive/ Negative

Please put a tick on your concerned line

Product name	Never experienced	Seldom used	Per day consumption				Per week consumption	Per month consumption
			150-200 mL	200- 350 mL	350-500 mL	>500 mL		
Sunflower								
Soyabean								
Canola								
Olive								
Corn								
Mustard								

Consent: I give my full consent to use my information for research purpose

First Name: ----------------------- **Signature/Date**: ---------------------
Middle Name: ----------------------
Family Name: ---------------------

Education:

Cell No:
